# “Taking the Snail for a Walk”: A Multi‐Stakeholder Qualitative Study Exploring Chinese Parents' Feeding Experiences With Preschool Children

**DOI:** 10.1111/mcn.70235

**Published:** 2026-07-28

**Authors:** Jian Wang, Yan‐Shing Chang, Xiaoxue Wei, Yang Cao, Kirsty Winkley

**Affiliations:** ^1^ Florence Nightingale Faculty of Nursing, Midwifery and Palliative Care King's College London London UK; ^2^ School of Nursing, Li Ka Shing Faculty of Medicine The University of Hong Kong Hong Kong SAR China; ^3^ Department of Hematology and Oncology Shanghai Children's Medical Center Affiliated to Shanghai Jiao Tong University School of Medicine Shanghai China; ^4^ Clinical Epidemiology and Biostatistics, School of Medical Sciences, Faculty of Medicine and Health Örebro University Örebro Sweden; ^5^ Unit of Integrative Epidemiology, Institute of Environmental Medicine Karolinska Institutet Stockholm Sweden

**Keywords:** feeding practices, parents, preschool children, qualitative

## Abstract

Childhood overweight and obesity are major global health concerns. Parents significantly influence children's eating behaviours and weight through feeding practices. In China, limited qualitative research explores parents' lived experiences in this context. This study aimed to examine Chinese parents' feeding experiences, challenges, coping strategies, and support needs in the home environment. A purposive sampling strategy was employed. Qualitative data were collected through semi‐structured interviews and focus groups with parents (*n* = 35), kindergarten teachers (*n* = 16), and healthcare professionals (*n* = 11) in China. Data were analysed using reflexive thematic analysis. The Family Systems Theory and Ecological Systems Theory informed the interpretation of the findings. Four key themes were identified: (1) feeding as a dynamic, phased, and adaptive process; (2) feeding can be challenging; (3) multiple feeding strategies as a response to challenges; and (4) opportunities for better supporting parents and families. Parents described an evolving process of feeding, marked by a shift from parent‐led approaches toward more balanced and adaptable routines. They encountered challenges, including limited time and knowledge, high expectations, family conflict, and insufficient support. In response, they employed diverse strategies such as acquiring greater knowledge, reflecting on practices, and seeking social support. Many parents also highlighted the need for trustworthy information, professional guidance, structured learning opportunities, and peer support. This study underscores the dynamic and multifaceted nature of parental feeding practices and calls for targeted, contextually appropriate interventions to support Chinese parents in fostering children's healthy eating habits during early childhood.

## Summary

- Parents' feeding experiences in Chinese home settings remain underexplored despite their central role in shaping preschool children's eating behaviours.
- Feeding practices change over time as parents respond to children's growing autonomy, family routines, cultural expectations and practical constraints.
- Parents need trustworthy, accessible and ongoing guidance on healthy feeding throughout the preschool years.
- Family‐centred interventions should involve healthcare professionals and early‐years educators to provide consistent support across home, healthcare and educational settings.

## 1 Introduction

Childhood overweight and obesity remain pressing concerns globally. These conditions often track into adulthood and are associated with an increased likelihood of developing chronic illnesses (Geng et al. 2018). In 2020, 7% of Chinese children under six were overweight and 3.6% obese, placing China among the countries with the largest child obesity populations (Pan et al. 2021). Multiple risk factors at various levels have been identified as contributors to childhood overweight and obesity, including genetics, epigenetics, behavioural patterns, and environmental factor (Silventoinen et al. 2010; Spruijt‐Metz 2011). Among these, the family food environment is particularly influential (Rosenkranz and Dzewaltowski 2008). As primary agents within this environment, parents have considerable involvement in children's dietary intake, eating behaviours and weight status through feeding practices (Faith et al. 2004; Ruzicka et al. 2021). Feeding practices refer to strategies that caregivers employ to manage what, when and how much their children eat and shape their children's eating behaviours (Vaughn et al. 2016; Wang, Zhu, Wu, et al. 2022). It is known that feeding practices have several dimensions, which can be generally categorised into two types: non‐responsive feeding (e.g., pressure to eat) and responsive feeding (e.g., modelling) (Savage et al. 2017; Wang, Zhu, Wu, et al. 2022).

To date, quantitative methods have been predominantly used to understand the factors, relationships, and outcomes associated with feeding practices (Costa et al. 2022; Ruzicka et al. 2021). Only a few qualitative studies have explored parental feeding experiences with children who have eating issues (e.g., picky eating) (Chilman et al. 2023; Edwards et al. 2024) or live in disadvantaged circumstances (So et al. 2024). Some other qualitative studies have explored feeding practices in specific cultural contexts (Momin et al. 2014; Mosli et al. 2019; Zhou et al. 2015). For example, in the United States, qualitative research suggested that Chinese immigrant mothers of preschoolers were using both known feeding practices (e.g., reward, monitoring) and less documented culturally‐emphasised practices (e.g., spoon‐feeding) (Zhou et al. 2015). Such studies highlight the fact that parental feeding practices often reflect cultural values and regional differences.

It is known that parents in China, particularly those within low‐ to middle‐income areas, often overfeed children due to the misconception that a higher body weight signifies better nutrition (Wang et al. 2025; Wang, Zhu, Cheng, et al. 2022). Additionally, non‐nuclear Chinese households frequently involve three‐generational caregiving, with grandparents playing an active role in child‐rearing (Jiang et al. 2007; Li et al. 2015). Influenced by ‘a good appetite is a blessing’, grandparents usually encourage overfeeding or indulgent practices, presenting a challenge to parents (Jiang et al. 2007). Given the lack of in‐depth research which has explored parents' feeding experiences with preschoolers in China, research is urgently needed to contribute culturally specific insights to the evidence base.

Paediatricians, nurses, and nutritionists all play a key role in monitoring children's growth and offering feeding‐related guidance during routine care, placing them in a unique position to identify parents' common challenges (de Pooter et al. 2022). Similarly, kindergarten teachers, who interact with children closely, can observe eating and weight changes and contribute insights into how parental feeding practices manifest in children's everyday routines (Chisholm et al. 2022). Indeed, these stakeholders can offer unique perspectives on contextual challenges, practical solutions, and the cultural norms that parents face (Story et al. 2008). However, few studies of parental feeding practices have included the viewpoints of healthcare professionals (HCPs) and kindergarten teachers. Given this, this qualitative study aimed to explore parents' feeding practices for preschoolers in the Chinese family context to gain a deeper understanding of their experiences, challenges, strategies and needs, incorporating perspectives from diverse stakeholders.

## 2 Methods

### 2.1 Study Design

This exploratory qualitative study was guided by a constructivist paradigm (Table S1), which posits that knowledge is constructed through interactions between individual experiences and their context (Madill et al. 2000). An experiential orientation was also considered as it emphasises participants' contextually situated experiences and perspectives (Braun and Clarke 2013).

We employed two theoretical perspectives, the Family System Theory (Bowen 1966) and Bronfenbrenner's Ecological Systems Theory (Bronfenbrenner 1979) to obtain a holistic understanding of Chinese parents' feeding experiences with preschoolers within the family environment. Family System Theory conceptualises the family as an interconnected unit in which individual members (subsystems) are interdependent. It emphasises the need to view the family as a whole to understand intra‐familial interactions, such as parent‐child dynamics. It was selected as it offers a comprehensive framework to explore parental feeding experiences within the context of family relationships. Ecological Systems Theory situates parents and children within evolving, interconnected social contexts (microsystem, mesosystem, exosystem, macrosystem, chronosystem) that shape development. This framework guided data interpretation and identified determinants of parental feeding practices, including related challenges, strategies and needs. The study reporting followed the Consolidated Criteria for Reporting Qualitative Research (COREQ) checklist (Tong et al. 2007) (Table S2).

### 2.2 Settings, Recruitment and Participants

Chinese kindergartens enrol children aged 2–6, divided into four stages: nursery (aged 2–3), pre‐kindergarten (aged 3–4), junior kindergarten (aged 4–5) and senior kindergarten (aged 5–6). Four public kindergartens in Yangzhou City, within the Jiangsu Province of Eastern China, were selected based on existing research collaborations (Wang et al. 2025) and geographical location. Purposive sampling recruits participants with relevant knowledge or experience who are available, willing and able to communicate effectively (Palinkas et al. 2015). Purposive sampling was used in this study to obtain a sample with diverse characteristics, targeting parents (e.g., age, education level), kindergarten teachers (e.g., working role, education level) and HCPs (e.g., professional role, years of experience). This approach was intended to ensure variation in participants' experiences and enhance the depth and richness of the qualitative data in relation to the study aims.

The inclusion criteria for parents were: (1) being responsible for their children's eating and the home food environment, (2) with at least one child aged 2–6 years, (3) being aged over 18 years, (4) being able to provide written informed consent, (5) being proficient in Chinese to participate in interviews. The exclusion criteria for parents were: (1) a diagnosis of severe physical and/or mental illness, such as cognitive impairment, and (2) having children with medical conditions that influence their eating and nutrition (e.g., diagnosed eating disorders).

The inclusion criteria for kindergarten teachers included: (1) having at least 3 years of kindergarten work experience, (2) holding a role related to the education, nutrition, and growth of preschool children, (3) being aged over 18 years, (4) being able to provide written informed consent, and (5) being proficient in Chinese.

For HCPs, the criteria were: (1) being able to provide written informed consent, (2) being proficient in Chinese, (3) being aged over 18 years, and (4) working in related areas such as nutrition, child education, or paediatrics.

After permission was provided by gatekeepers, parents and teachers were recruited through posters displayed on kindergarten notice boards. Participant information sheets were distributed during parent meetings by teachers. HCPs were recruited through visits in hospitals by the lead author (JW) and emails with invitation posters and participant information sheets. Interested participants contacted JW, were screened for eligibility, and then asked to sign a consent form.

### 2.3 Data Collection

Semi‐structured interviews were held with parents and HCPs, and focus groups were held with teachers, between April 2023 and July 2023. All interviews and focus groups were conducted by JW. Informed by our previous academic work (Wang et al. 2024; Wang, Zhu, Wu, et al. 2022; Wang, Zhu, Cheng, et al. 2022) and relevant qualitative studies (Galvez Espinoza et al. 2022; Mosli et al. 2019; Zhou et al. 2015), a topic guide was established for parents, teachers and HCPs (Table S3). The questions focused on: (1) parents' experiences with feeding preschoolers; (2) the feeding challenges and barriers encountered; (3) potential strategies to improve feeding; and (4) perceived needs. The topic guides were refined after piloting the interviews and checking with the research team (KW, Y‐SC and YC).

Participants were offered a choice between face‐to‐face and telephone interviews. Face‐to‐face semi‐structured interviews and focus groups were conducted in quiet rooms at the kindergartens. Other interviews were conducted via telephone. All interviews were audio‐recorded with participants' consent. Field notes were taken throughout to capture JW's insights and guide subsequent interviews and focus groups.

### 2.4 Data Analysis

**Figure 1:**
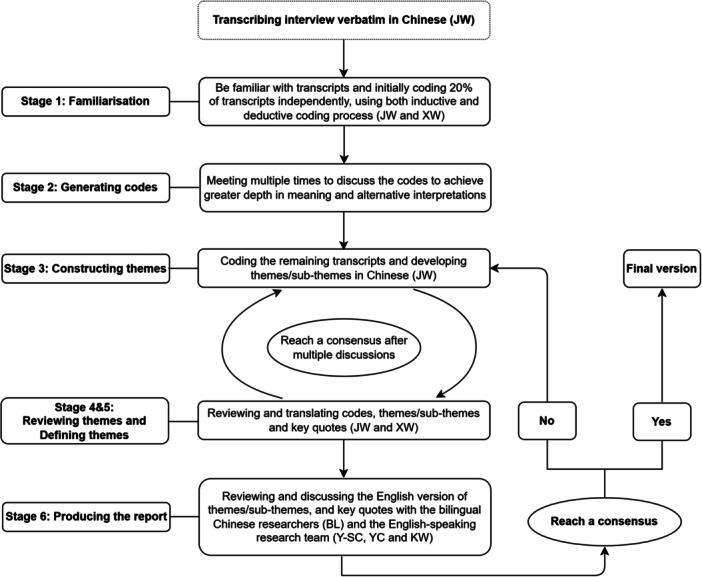
The collaborative and iterative data analysis and translation procedure.

We used reflexive thematic analysis, a theoretically flexible method which facilitates the development, analysis, and interpretation of meaning within data (Braun and Clarke 2022). Individual interviews and focus groups were transcribed, pseudonymised, and automatically translated using iFLYTEK software, then manually checked for accuracy by JW. NVivo 14 was employed to organise data, make memos, and support coding and analysis. An iterative and collaborative analysis and translation procedure was implemented (Braun and Clarke 2006, 2022; Douglas and Craig 2007). A schematic overview of this procedure is presented in Figure 1. Data from kindergarten teachers and HCPs were used to provide contextual understanding and triangulate parental accounts, rather than to conduct formal comparative analysis across stakeholder groups. The interviews and focus groups were coded independently by two authors (JW and XW) using inductive and deductive coding processes (Table S1). By employing both coding approaches, we aimed to uncover the latent (hidden) and semantic (explicit) meanings within the data. All generated themes/sub‐themes, codes and key data extracts were checked by XW (a female bilingual researcher with expertise in nursing, child feeding, and qualitative research). They were then discussed with the research team (Y‐SC, KW and YC) and a bilingual Chinese researcher (BL).

### 2.5 Rigour

The trustworthiness criteria developed by Lincoln and Guba were considered during the entire research process (Lincoln and Guba 1985). Credibility was achieved by building up a research team with different cultural backgrounds and multiple specialities (i.e., nursing, child health and nutrition, education, and epidemiology) to obtain a more in‐depth understanding of the data. Dependability was ensured through a detailed methodological description: two independent researchers performed and reviewed the codes and themes, and all researchers participated in the discussion about the final themes and subthemes. Confirmability was assured by maintaining an audit trail of coding decisions and researcher reflexivity (e.g., field notes). Transferability was judged by the description of the study context (i.e., Chinese home food environment) and participants' characteristics.

### 2.6 Reflexivity

The interviewer (JW, female, PhD) had a background in early childhood nutrition and parental health behaviours, with prior experience conducting qualitative research. Being local to the study site, JW was well positioned to establish rapport with participants and interpret cultural nuances. Reflexive awareness was also maintained by employing open‐ended prompts and avoiding preconceived assumptions. Open coding was used to inductively capture participants' perspectives. Insights from previous academic work and relevant theoretical frameworks informed the interpretation process, enhancing analytical rigour without imposing theoretical constraints on the data.

### 2.7 Ethical Considerations

This study received approval from the Research Ethics Committee at King's College London (HR/DP‐22/23‐35316) and the Institutional Review Board at Baoying Maternal and Child Health Hospital in Yangzhou, China (YZBFYLL‐202303). Written informed consent was obtained from all participants prior to data collection.

## 3 Results

### 3.1 Sample Characteristics

In total, 62 participants were interviewed: 35 parents, 16 teachers and 11 HCPs (Table 1). The mean age of parents and children was 32.69 years and 46.91 months respectively. Most included caregivers were mothers (*n* = 29), and nearly half of parents had two or more children. The mean years of experience for teachers and HCPs were 10.69 (range 3–28) and 10.7 (range 3–27), respectively. Of the 73 potential participants who expressed interest, four were ineligible and seven declined to participate. The duration of interviews ranged from 29 to 58 min. Focus groups lasted between 62 and 77 min.

**Table 1:** Sample characteristics.

Characteristics of parents	*N* = 35	Characteristics of kindergarten teachers	*N* = 16	Characteristics of HCPs	*N* = 11
Parents age (years) Mean (range)	32.69 (24–48)	Age (years) Mean (range)	34.94 (24–51)	Age (years) Mean (range)	38.5 (28–55)
The role of parents		Working roles		Sex	
Mother	29	Class teacher	10	Female	10
Father	6	Childcare teacher	4	Male	1
		Grade supervisor	2	Educational level	
Parental weight status (self‐reported)		Years of working Mean (range)	10.69 (3–28)	College or higher	8
Underweight	4	Educational level		Junior college	3
Normal weight	17	College or higher	7	Professional role	
Overweight/obese	14	Junior college	9	Physician	4
Educational level		Class Tier		Registered nurse	4
Senior high school or below	14	Nursery or Toddler Class	2	Early childhood educator (policy maker)	1
Junior college	10	Pre‐Kindergarten	4	Academic researcher	2
College or higher	11	Junior Kindergarten	5	Professional area	
Employment status		Senior Kindergarten	5	Child education	2
Full‐time work	18	Sex		Social science	1
Part‐time work	5	Female	16	Paediatrician	2
Self‐employed	2	Focus groups		Dietitian	2
Full‐time parents	10	Face‐to‐face	16	Nursing	4
Number of children				Interview modes	
One	18			Face‐to‐face	7
Two or more	17			Virtual	4
Child age (months) Mean (range)	46.91 (24–72)			Years of experience Mean (range)	10.7 (3–27)
Child sex				Work locations (province)	
Boy	17			Jiangsu	7
Girl	18			Shanghai	2
Child weight				Zhejiang	2
Underweight	7				
Normal weight	18				
Overweight	6				
Obese	4				
Family structure					
Parents and grandparents	22				
Only parents	13				
Interview mode					
Face‐to‐face	25				
Virtual	10				

### 3.2 Thematic Findings

**Figure 2:**
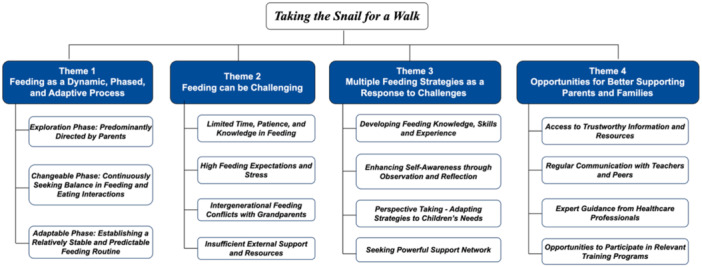
Study themes and subthemes.

Four main themes and accompanying subthemes were developed (Figure 2). Exemplar quotes are presented in Table 2. Theme 1 is based on parents' accounts of lived feeding experiences, whereas Themes 2–4 integrate perspectives from parents, kindergarten teachers and HCPs to contextualise parents' feeding challenges, strategies and support needs.

**Table 2:** Themes, subthemes, and example quotes reflecting parents' experience of feeding practices with preschoolers.

Themes	Subthemes	Example quotes
Feeding as a dynamic, phased, and adaptive process	1.1 Exploration phase: predominantly directed by parents	# 1 ‘I think children's eating behaviours vary significantly by age group. Younger children, in particular, have limited autonomy and require more guidance and supervision from parents and teachers, as they have not yet developed healthy eating habits’. (*FG1/T6 Lead teacher*) # 2 ‘If my child ate more than usual and wanted more, I generally didn't give extra because I worried about overeating. This decision was based on my intuition since I wasn't sure of her appetite and she couldn't clearly express, only saying ‘no, no’ when she didn't want to eat anymore’. (*P13 Father*) # 3 ‘It wasn't too early for them to be picky eaters. I thought other eating issues, such as talking while eating, tended to emerge as they got older. At her current age (24 months old), my priority was getting her to sit quietly and eat independently’. (*P5 Mother*) # 4 ‘I offered my children various foods, similar to what the adults were eating, but I noticed they didn't like rough or hard‐textured foods, so I adapted their meals accordingly’. (*P12 Father*) # 5 ‘My child (aged 26 months) should have started eating independently, but she didn't cooperate unless she was hungry. Since she was still young, sometimes I satisfied her needs for spoon‐feeding’. (*P14 Mother*)
	1.2 Changeable phase: continuously seeking balance in feeding and eating interactions	# 6 ‘At first, I spoon‐fed my child and then gradually taught her to eat by herself. By 46 months old, she was capable of eating independently’. (*P22 Mother*) # 7 ‘Forcing him to eat used to work before, but now he refuses and spits out food. I don't understand his pickiness. I'm still considering approaches to address this’.(*P21 Mother*) # 8 ‘Sometimes we tried to sneak vegetables into my child's meals by chopping them very finely, so he didn't notice them'. (*P24 Mother*) # 9 ‘We made improvements to his feeding habits. I hope he eats more vegetables. I talked to him about the benefits of a healthy diet and used his cousin as an example. Sometimes he resisted, but I would explain the importance of eating for growth. This method did work’. (*P18 Mother*) # 10 ‘My child had little interest in eating. I tried various strategies to improve his habits, but none seemed effective. He often begged for snacks, saying “I'm starving, I want some snacks.” Withholding snacks was too strict, but giving in would affect his meals. I was still struggling to find a solution’. *(P33 Mother*)
	1.3 Adaptable phase: establishing a relatively stable and predictable feeding routine	# 11‘Feeding my child has mostly been worry‐free; she ate independently and stopped when full. I seldom pressured her to eat all. However, a problem arose with her inability to self‐regulate eating. Without supervision, she continuously ate as if she was never satisfied. I started to moderately restrict her snack times. Other than this, her eating habits were well‐balanced’. (*P23 Mother*) # 12 ‘I didn't set strict eating rules or pressure my child to eat, but I did encourage her. Deep down, I hoped she would eat, but if she didn't want to, that was fine. I never forced her to eat because she knew when she was hungry. Overeating could lead to indigestion. If she didn't feel like eating, she might just sip some soup in the evening. I thought this helped her regulate her digestive system’. (*P2 Mother*) # 13 ‘When my child was younger, if he ate slowly or got distracted, I tried different approaches to encourage his eating, but none worked. Eventually, I realised that children had own eating paces and forcing to eat doesn't help. Instead, I focused on supervising and gently prompting him, and I reminded myself to be patient. Shifting my mindset helped me accept and adapt to his eating habits’. (*P32 Mother*)
Feeding can be challenging	2.1 Limited time, patience, and knowledge in feeding	# 14 ‘I didn't have enough time to monitor my child's eating closely; grandparents helped with feeding. My child, over two years old, couldn't eat independently and would become hysterical during meals. I was confused and impatient, unsure how to address it. I felt that if he ate something, it was generally good (Sigh)’. (*P20 Mother*) # 15 ‘I struggled to understand how to feed my child effectively and lacked professional knowledge in this area. It's still a challenge to correct his poor eating habits. My methods couldn't compare to those of professionals, and I often doubted whether they were good’. (*P11 Mother*) # 16 ‘I believe that change would only happen if parents recognised feeding issues as serious. Many parents lacked the necessary knowledge, and some thought children would naturally outgrow unhealthy eating habits’. (*FG3/T12 Class teacher*) # 17 ‘Busy schedules and pressure made it hard for parents to address feeding issues consistently. Overindulgence also made feeding increasingly difficult, and parents quickly became exhausted and didn't persist in guiding children'. (*H1 Researcher in early childhood education*)
	2.2 High feeding expectations and stress	# 18 ‘I was truly anxious, and honestly, I wasn't sure what to do. Every parent, if asked, would have expressed a desire for their child to be taller, heavier, and eating well. I hoped he would eat everything and look chubby and healthy. However, my child showed little interest in food. It was a worry’. (*P33 Mother*) # 19 ‘While other kids seemed to eat well, my child wasn't interested in food, which truly worried me. Who wouldn't want their child to be healthy and chubby? I tried to correct his picky eating. However, feeding nowadays isn't as straightforward as it used to be. What should I do?’ (*P21 Mother*)
	2.3 Intergenerational feeding conflicts with grandparents	# 20 ‘My child is overweight and tends to overeat. Despite my efforts to control her snacking, her grandmother often intervened, leading to my child becoming dependent. She would always seek out her grandmother for indulgence whenever she wanted to eat’. (*P10 Father*) # 21 ‘I tried to stop the grandparents from spoon‐feeding the child, but it didn't work. I wanted to correct my child's unhealthy eating habits, but the involvement of grandparents increased feeding difficulties’. (*P1 Mother*) # 22 ‘I often heard the phrase “grandparents spoil the children.” Many grandparents indulged their grandchildren, even when parents disagreed. The older generation believed their experience was valuable and hard to change’. (*FG2/T10 Childcare teacher*) # 23 ‘When children came in for check‐ups, we always asked about feeding and eating. It was easier to communicate with parents, but with grandparents, they often didn't grasp it. They didn't see their approaches as wrong; rather, they believed they did a great job with childcare. Our healthcare professionals provided feeding advice but weren't very promising as implementation was minimal’. (*H5 Paediatric nutritionist*)
	2.4 Insufficient external support and resources	# 24 ‘The online information I found was only somewhat helpful. Every child is different, so specific recommendations would have been more useful. Overall, I found the online information lacked expertise’. (P36 Mother) # 25 ‘I wanted to connect with other parents and friends, but we were all busy, and there were only a few parent‐teacher meetings each semester. I felt it was essential to discuss eating issues with other parents of my child's classmates, but such conversations seldom happened’. (P24 Mother) # 26 ‘It was difficult to see a healthcare provider or nutritionist regularly due to my work commitments. The annual check‐up mainly focused on child's growth, and there wasn't much time for detailed guidance on child feeding. Outpatient clinics were usually quite busy’. (*P23 Mother*) # 27 ‘We asked parents briefly about their child's eating at routine clinic checkups. Due to the large number of children in the clinic and limited time, we could not inquire in detail about each child. Some parents didn't care about feeding as long as their child was healthy, so additional advice was not needed’. (*H6 Paediatrician*)
Multiple feeding strategies as a response to challenges	3.1 Developing feeding knowledge, skills and experience	# 28 ‘I used to study feeding knowledge online related to how to teach children to eat independently. I found some advice useful, such as not forcing children to eat more and helping establish healthy mealtime rules. I also bought a book on scientific feeding recipes, which I sometimes read to learn more’. (*P14 Mother*) # 29 ‘There was a change! Parenting my first child was challenging due to my lack of experience. With my first child, even small issues made me nervous. However, with my second kid, things went more smoothly, and I knew these difficulties would eventually be resolved. My younger child was picky about food, I knew this was normal because I noticed that as children get older, they become open to trying new foods. I didn't force her to change and now she has greatly improved’. (*P2 Mother*) # 30 ‘Some parents had various obstacles while parenting their first child, but these experiences prepared them to handle their second as they knew a lot about their children's development and eating habits. Parents often strictly adhere to rules and books with their first child, but with the second, they tend to have a better attitude and do not feel overwhelmed’. (*FG2/T11 Childcare teacher*)
	3.2 Enhancing self‐awareness through observation and reflection	# 31 ‘I had a friend whose child was thin and struggled with eating, often taking over an hour for a meal. I noticed her grandmother primarily fed her, giving her a bite of rice followed by a sip of soup. I think that's why this child had poor chewing skills. Seeing their feeding issues, we let my child eat independently and I reminded him to chew properly and focus on eating’. (*P6 Mother*) # 32 ‘Children's eating habits at school often mirror their family's dietary environment. Their behaviour reflects their parents' lifestyle and attention to eating habits. Some parents, after self‐reflection, recognised the need for change and sought professional support or communicated with our teachers’. (*FG2/T9 Class teacher*) # 33 ‘Some parents adjusted their mindset, which benefited their children. Significant improvements include many people being more patient. Real change occurs when parents themselves acknowledge the need for change and act’. (*H2 Paediatric nurse)*
	3.3 Perspective taking: adapting strategies to children's needs	# 34 ‘I felt if food was chopped up, my child could eat almost anything. Considering his young age with a narrow throat, we need to pay attention to these physiological traits. For example, salmon didn't need fine chopping as it's naturally tender. We used minimal seasonings and offered a variety of foods, like alternating between salmon and cod, to make meals interesting’. (*P19 Father*) # 35 ‘You should tailor your methods to your child's needs. My child used to eat slowly, and as a former picky eater with a laid‐back personality, I understood her. I encouraged her to try new foods without pressuring her. I disagreed with her father's approach of criticising without explanation. From my experience of being criticised for not eating well, it was upsetting for a child’. (*P17 Mother*) # 36 ‘I believe it is essential to put yourself in your child's shoes. Just telling them not to do is often unsuccessful owing to their lack of understanding and self‐control. Parents need invest time and effort in shaping children's healthy eating habits. Direct demands or enforcing rules without explanation are ineffective; young children need to know how to act’. (*H7 Researcher in child health and nutrition*)
	3.4 Seeking powerful support network	# 37 ‘Perhaps some people around me were more knowledgeable and professional, such as our neighbour who was a paediatrician, and grandmother who attended some childcare training and provided me with sensible feeding advice’. (*P5 Mother*) # 38 ‘Talking with friends about my child's eating was helpful. My child struggled to focus during meals. Some friends had already been through this, while others were in the same situation, facing different feeding challenges. Sharing experiences eased my stress, and I learned useful strategies from them’. (*P14 Mother*) # 39 ‘As teachers, our main role is to guide, observe, and encourage the children. I also communicated with parents about kids who were easily distracted and played during meals, explaining the issues and sharing strategies to correct these habits. With parental support, we usually saw improvements. Collaboration between home and school is crucial’. (*FG3/T15 Lead teacher*) # 40 ‘If parents guided their children well from an early age, diet issues were rarely a concern. Some children in our clinic have weight problems. We started with a medical assessment to rule out organic disorders, then provided guidance, such as establishing mealtimes, and reducing distractions. We gave parents a letter with the results and specific advice after the check’. (*H8 Paediatrician*)
Opportunities for better supporting parents and families	4.1 Access to trustworthy information and resources	# 41 ‘I often browsed online and paid attention to relevant content, but I couldn't tell if it was accurate. It would be great if there were trustworthy resources available, such as guideline on what foods children of certain ages should consume regularly. Ideally, weekly template and recipes would help me plan meals better’. (*P26 Mother*) # 42 ‘The Chinese Nutrition Society published a preschool nutrition guide in 2022 with a dietary pyramid, offering parents useful feeding advice. However, I found that these materials were not widely distributed. Some apps to track children's growth and eating were also easy to use. Only a few parents used these tools, I suppose’. (*H9 Dietitian*)
	4.2 Regular communication with teachers and peers	# 43 ‘As a new parent, I realised I could improve through peer communication. Conversations helped me see where I can improve. I seldom had the chance to discuss parenting approaches because few parents in my social circle had similar conditions. I need to connect to others and learn from their methods’. (*P17 Mother*) # 44 ‘Many parents may not realise issues with their feeding methods. They often believe their child's eating is satisfactory. However, after talking with teachers, they recognised the seriousness. For example, children who refused to eat vegetables led me to discuss with their parents, revealing their food availability at home was very limited. Such exchanges are essential in helping parents become aware of feeding issues’. (*FG3/T16 Childcare teacher*)
	4.3 Expert guidance from healthcare professionals	# 45 ‘I was willing to adopt scientific feeding methods, as most of us relied primarily on personal experience or intuition. If I receive professional guidance, I believe it would help refine my feeding strategies, more targeted and effective’. (*P6 Mother*)
	4.4 Opportunities to participate in relevant training programmes	# 46 ‘Could we establish specific training on child feeding and nutrition for parents? There were no such courses. The suggestions provided online were not ideal, and children were not well accepted. As parents, we need to understand this aspect of knowledge systematically and in detail’. (*P16 Mother*) # 47 ‘Training development is essential, particularly in multi‐module courses. By offering training, parents can gain knowledge and understand why such practices are necessary. Moreover, parents would have the opportunity to sit together, discuss, and share experiences. However, until now, there were no training programmes targeted at parents in kindergartens’. (*H3 Early childhood educator*) # 48 ‘Our kindergarten did not have specific courses aimed at parental feeding, but they were indeed necessary. I think kindergarten should organise at least one event per year for parents. More materials, such as educational videos, can be distributed to enhance parents' knowledge and skills via social media. Furthermore, engaging parents may generate a ripple effect, disseminating feeding knowledge’. (*FG1/T2 Class teacher*)

Abbreviations: FG, focus group; H, healthcare professionals; P, parent; T, teacher.

### 3.3 Theme 1 Feeding as a Dynamic, Phased, and Adaptive Process

Many parents described a continuous cycle of adjusting feeding approaches in response to children's changing food responses, nutritional needs, and developing autonomy across the preschool years, which aligns with Family Systems Theory. Rather than representing fixed feeding practices, these accounts suggested a dynamic, phased and adaptive process in which parents gradually moved from more parent‐directed feeding towards greater responsiveness to children's emerging preferences, abilities, and self‐regulation.

Feeding is complex. Parents learn and adapt over time to find approaches that suit their preschool child.

(P34, Mother)

The metaphor ‘taking the snail for a walk’, offered by an early childhood educator, contextualised a broader pattern in parents' accounts: feeding was experienced as a slow, uncertain, and relational process requiring patience, responsiveness, and continual adaptation to children's developing needs, preferences, and autonomy.

Feeding takes time and requires understanding children's needs, intrinsic motivation, and a gentle approach that fosters independence. There is no universal model; parents must adapt, like taking a snail for a walk: slow, patient, and caring.

(H1 Researcher in early childhood education)

#### 3.3.1 Subtheme 1.1 Exploration Phase: Predominantly Directed by Parents

Thirteen parents of 2‐ to 3‐year‐olds described this phase as a transition after breastfeeding and complementary feeding. Children began exploring new foods and textures, but limited autonomy and communication (*quote 1*) made needs hard to express. Parents therefore relied on intuition, knowledge, and past experience to guide feeding, largely directing their child's eating (*quote 2*).

Parents perceived children's eating at this stage as relatively dependent on parental guidance, although early signs of individual food preferences and picky eating began to appear (*quote 3*). When this happened, many parents adjusted their feeding strategies based on their children's responses, for example to ‘rough or hard‐textured foods’ (*quote 4*). As self‐feeding skills were limited, many parents spoon‐fed children, especially when they resisted eating (*quote 5*). At this stage, parental control was often shaped by children's limited communication and self‐feeding abilities, meaning that parents had to interpret children's cues and make feeding decisions on their behalf.

#### 3.3.2 Subtheme 1.2 Changeable Phase: Continuously Seeking Balance in Feeding and Eating Interactions

Ten parents of preschoolers aged 3–5 were at this phase. Whilst some parents reported improved self‐feeding (*quote 6*), others noted challenging behaviours (e.g., slow eating, low food interest) that, though typical of development, often left them confused and frustrated (*quote 7*). This phase reflected a more visible tension between parental expectations around nutrition and children's emerging preferences, requiring parents to adjust boundaries, routines, and feeding strategies. Many parents adapted feeding strategies, such as chopping or hiding vegetables, to accommodate children's eating patterns (*quote 8*).

As children encountered diverse foods, their preferences became more defined while still flexible. Parents sought to balance these preferences with their own expectations, requiring learning, support, and adjustments within the family environment. Some parents established feeding routines suited to their child (*quote* 9), whereas others were still experimenting with different strategies, including withholding snacks, to manage feeding challenges (*quote* 10).

#### 3.3.3 Subtheme 1.3 Adaptable Phase: Establishing a Relatively Stable and Predictable Feeding Routine

Twelve parents of 5‐ to 6‐year‐olds in the final kindergarten year were navigating this phase. At this stage, children generally exhibited more stable or ‘well‐balanced’ eating habits (*quote 11*). Many parents became more skilled at recognising children's preferences and gradually clarified feeding roles, encouraging self‐regulation rather than forcing eating (*quote 12*). Parents' growing expertise appeared to enable them to establish stable, predictable family feeding routines. Some parents also experienced periods of profound self‐reflection and adaptation, including ‘reminding themselves to be patient’, recognising that ‘forcing to eat doesn't help’, and consciously ‘shifting mindset’ to better adapt to their children's eating habits (*quote 13*). This is a gradual shift from direct control towards guidance, patience, and support for children's self‐regulation.

### 3.4 Theme 2 Feeding Can Be Challenging

Feeding challenges arose from individual, family, and external social factors, aligning with the multiple levels described in Ecological Systems Theory. Nearly all parents reported difficulties, with many expressing confusion and anxiety:

He was picky, preferring fried skewers, sausages, and chips. He called vegetables ‘leaves’ and refused them. Despite trying different strategies, none worked, and I often felt helpless managing his difficult eating.

(P7 Mother)

#### 3.4.1 Subtheme 2.1 Limited Time, Patience and Knowledge in Feeding

Balancing work and parenting often left parents short of time and patience, leading to compromises when facing children's challenging eating behaviours (*quote 14*). Some parents reported limited knowledge and doubts about their methods, leading to inappropriate strategies and reduced confidence in shaping children's eating habits (*quote 15*).

Furthermore, some teachers and HCPs believed that parents had little awareness of how ‘serious’ feeding issues were or thought that they would just ‘outgrow’ unhealthy eating, and hence did not invest time or effort to address them (*quote 16*). Parents were sometimes perceived as indulging their children's unreasonable dietary needs to simplify feeding challenges (*quote 17*).

#### 3.4.2 Subtheme 2.2 High Feeding Expectations and Stress

Parents set various feeding expectations, often hoping their child would eat enough to appear ‘chubby’ and ‘healthy’. When children's eating failed to meet expectations, parents experienced considerable stress and anxiety (*quote 18*). Some parents reported that stress made it harder to sustain effective feeding strategies (*quote 19*).

#### 3.4.3 Subtheme 2.3 Intergenerational Feeding Conflicts With Grandparents

Many parents reported intergenerational feeding conflicts with grandparents, particularly when grandparents' feeding beliefs and practices differed from parents' efforts to establish consistent feeding habits (*quotes 20–21*). Parents described grandparents' feeding beliefs as ‘outdated and difficult to change’, noting they often overindulged children. This was reflected in grandparents spoon‐feeding or giving children whatever they wanted to eat. Kindergarten teachers and HCPs noted co‐parenting conflicts, which significantly contributed to feeding challenges (*quotes 22–23*).

#### 3.4.4 Subtheme 2.4 Insufficient External Support and Resources

Several parents reported inadequate support in resolving feeding difficulties. Some spoke about the lack of specificity or ‘expertise’ of online resources available (*quote 24*). Some parents wanted more peer support to ‘connect with other parents and friends’ for practical assistance (*quote 25*). Furthermore, some parents found it challenging to access professional advice *(quote 26)*. Parents and HCPs noted insufficient time during routine clinic visits for in‐depth discussions on feeding strategies (*quote 26–27*).

### 3.5 Theme 3 Multiple Feeding Strategies as a Response to Challenges

Children's eating habits are dynamic, prompting parents to adopt coping strategies across individual, family, and external support levels, reflecting the interconnected contexts described in Ecological Systems Theory. These strategies were not only practical responses to feeding challenges but also reflected parents' ongoing adjustments in parent–child feeding relationships through learning, reflection, support‐seeking, and responsiveness to children's needs. Kindergarten teachers and HCPs provided complementary perspectives on how such strategies were shaped.

Parents reported using multiple approaches to inform themselves, which other stakeholders also acknowledged:

Some parents were equipped with rich knowledge. They understand details even better than we do.

(H4 Early childhood educator)

#### 3.5.1 Subtheme 3.1 Developing Feeding Knowledge, Skills and Experience

Some parents spoke about expanding their knowledge and skills, such as ‘searching online’, buying ‘scientific feeding recipe books’ and participating in lectures and activities (*quote 28*). Parents often felt anxious with their first child's eating problems but, with accumulated expertise, relied less on books and guidelines for later children and felt better equipped to manage similar situations (*quotes 29–30*).

#### 3.5.2 Subtheme 3.2 Enhancing Self‐Awareness through Observation and Reflection

Parents learnt by observing others' feeding approaches (e.g., family members, caregivers), an important way to assess whether their own practices were appropriate. Some parents learnt by avoiding practices in their social networks (e.g., friends and neighbours) which they felt were inappropriate (*quote 31*). Kindergarten teachers and HCPs noted that self‐reflection helped parents recognise ‘the need for change’, such as being more patient or acknowledging the need for ‘professional support’ (*quotes 32–33*).

#### 3.5.3 Subtheme 3.3 Perspective Taking— Adapting Strategies to Children's Needs

Some parents clearly understood that it was important to ‘put yourself in your child's shoes’ and personalised their strategies to meet their child's needs. These included considering children's psychological and physiological traits by offering easy‐to‐swallow foods, varying textures and seasonings, and adopting approaches suited to their personality (*quotes 34–35*). As children grow, their dietary behaviours change. Participants, including professionals, stressed the importance of choosing appropriate timing and strategies for food education, using resources appealing across age groups (*quotes 36*).

#### 3.5.4 Subtheme 3.4 Seeking Powerful Support Networks

Several parents noted the benefit of seeking support from family, peers, and kindergarten teachers when facing feeding problems. They reported their social networks facilitated practical support, whilst ‘sharing experiences’ helped to relieve concerns and stress (*quotes 37–38*). Some kindergarten teachers advised parents on feeding problems they had observed at school (*quote 39*). HCPs highlighted their role in helping parents address concerns such as childhood obesity (*quote 40*).

### 3.6 Theme 4: Opportunities for Better Supporting Parents and Families

Stakeholders identified several opportunities for better supporting families with preschool child feeding. Parents primarily emphasised the need for accessible and trustworthy information, reassurance, and practical guidance. Kindergarten teachers highlighted the importance of communication, shared understanding, and consistency between home and kindergarten. Healthcare professionals placed greater emphasis on professional guidance, early identification of feeding concerns, and structured education or training opportunities. Together, these perspectives reflected the ecological nature of parents' support needs, suggesting that effective support should operate across family, educational, healthcare and community contexts.

When unsure about feeding issues, I felt stressed and questioned my approach. I sought professional advice from HCPs, then observed outcomes and requested feedback. This cycle of guidance and response felt more effective.

(P24 Mother)

#### 3.6.1 Subtheme 4.1 Access to Trustworthy Information and Resources

Parents raised diverse questions on feeding, including strategies, meal guidelines, and nutrient intake. Unless problems required professional referral, these concerns went unanswered, leaving parents anxious about the reliability of online information (*quote 41*). HCPs noted that although reliable online resources and nutritional guidelines exist to support family dietary routines (*quote 42*), parents did not always access them.

#### 3.6.2 Subtheme 4.2 Regular Communication With Teachers and Peers

New parents were keen to share and discuss strategies and dietary patterns with peers, although they were not always able to ‘connect’ (*quote 43*). Inappropriate feeding methods could become routine, with eating problems unacknowledged by parents. Some teachers noted that more regular communication with parents could prompt reassessment and improvement of feeding practices, avoiding ineffective strategies (*quote 44*).

#### 3.6.3 Subtheme 4.3 Expert Guidance From Healthcare Professionals

Many parents realised their feeding relied on ‘experience or intuition’ and sought greater professional guidance. Some parents noted that in‐depth conversations with healthcare providers helped identify feeding issues and generate personalised solutions tailored to their child's eating behaviours (*quote 45*).

#### 3.6.4 Subtheme 4.4 Opportunities to Participate in Relevant Training Programmes

Stakeholders suggested kindergarten‐based training could help parents acquire feeding knowledge and peer support. Parents also sought to improve abilities through ‘specific training on child feeding and nutrition’ (*quote 46*). Kindergarten teachers and HCPs noted that relevant training programmes in schools and communities were rare. However, they believed that ‘multi‐module’ training with relevant media could address inappropriate feeding practices and reach more parents, including those with limited access to professional guidance (*quotes 47–48*).

## 4 Discussion

This is the first qualitative study explored parents' experiences, challenges, strategies and needs regarding feeding practices within the study context in China, incorporating perspectives from diverse stakeholders.

As children's eating behaviours evolved during the preschool years, parents in our study engaged in a continuous process of adapting their feeding approaches. Our study contributes conceptually by framing preschool child feeding as a phased and relational process, building on existing work on the feeding relationship, responsive feeding, and food parenting practices (Hurley et al. 2011; Satter 1990; Vaughn et al. 2016). The three phases identified in the findings (i.e., exploration, changeability, and adaptability) suggest that parental feeding practices are not fixed behaviours, but evolve as children's eating abilities, preferences, autonomy, and communication develop (Savage et al. 2007). In the exploration phase, parents tended to take a more directive role because children's self‐feeding skills and ability to express needs were limited. In the changeable phase, parents increasingly adjusted their expectations and approaches in response to children's emerging food preferences, autonomy, and resistance. In the adaptable phase, many parents appeared to develop more stable routines and a clearer understanding of feeding roles, including encouraging children's self‐regulation rather than relying on force. This phased interpretation extends existing feeding literature by showing how parental feeding practices develop through a gradual balancing of parental responsibility and children's increasing autonomy. This interpretation is consistent with, and builds on, Satter et al.'s (Satter 1990) Division of Responsibility theory, which proposes that parents should determine what, when, and where food is served, while children should decide how much to eat and whether to eat. Our findings suggest that, in practice, parents' understanding and implementation of these feeding roles may develop gradually across the preschool years, as they learn to balance responsibility, control, and children's increasing autonomy.

**Figure 3:**
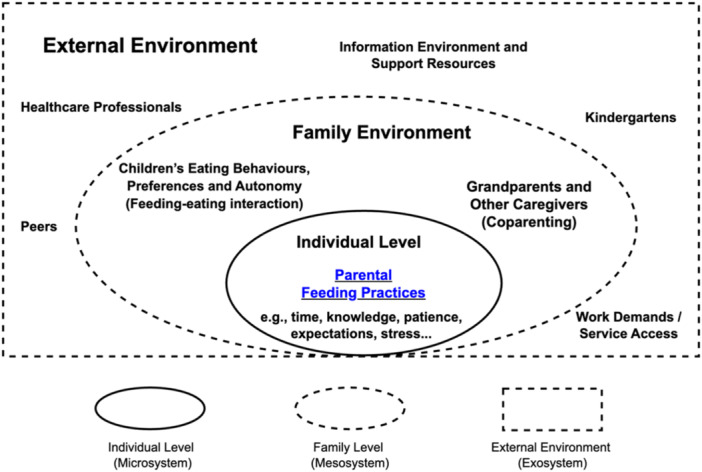
An ecological model of parental feeding practices.

Family Systems Theory and Ecological Systems Theory helped interpret feeding interactions, challenges, strategies and support needs across interconnected levels (Figure 3). Family Systems Theory helped interpret feeding practices within the family as an interconnected system, including parent‐child feeding dynamics, other caregivers' involvement, and intra‐familial collaboration or conflict. Ecological Systems Theory further situated these family processes within broader individual, family, and external contexts that shaped parents' feeding experiences. Specifically, feeding challenges and potential solutions stemmed from individual, family, and external factors. From an individual level, parents expressed their limited knowledge, time and patience, particularly when addressing children's challenging eating (e.g., low interest in eating). They also shared feelings of frustration and stress when children failed to meet their expectations (e.g., being a healthy child eater). These findings are supported by previous qualitative studies (Chilman et al. 2023; Edwards et al. 2024), which explored experiences of feeding preschoolers with eating problems (e.g., picky eating). For instance, an Australian study found that parents reported feelings of bitterness and guilt towards preschoolers' pickiness (Chilman et al. 2023). It also identified several strategies to manage children's picky eating (e.g., feeling empathetic and employing pragmatic responses). However, these individual‐level challenges should not be interpreted exclusively as parental limitations. Parents' time, access to reliable feeding information, and ability to seek professional support may also be shaped by broader structural and socioeconomic conditions, including work demands, inflexible schedules, caregiving arrangements, and access to professional support (Bauer et al. 2012; Story et al. 2008). Although gendered expectations around feeding did not emerge as a sufficiently consistent theme in the present dataset, broader evidence suggests that unpaid childcare and domestic work remain unevenly distributed between women and men in China (Dong and An 2015). Future research should therefore examine how changing labour patterns, parental work schedules, and gendered caregiving expectations shape feeding responsibilities and support needs among mothers, fathers, and other caregivers. Our study also found that parents employed diverse strategies to address feeding difficulties. Individually, parents equipped with sufficient knowledge and skills appeared more confident in handling challenges. Some enhanced self‐awareness through careful observation and reflection. This highlights the importance of personal factors (Savage et al. 2007), specifically the role of individual understanding, awareness, and strategic decision‐making, in optimising feeding practices and overcoming eating challenges.

At the family level, and consistent with Family Systems Theory and situated within the mesosystem of Ecological Systems Theory, many participants expressed concerns that grandparents' feeding practices were outdated, citing examples such as overindulgence and overfeeding. This aligns with previous studies in China (Jiang et al. 2007; Li et al. 2015) which found such conflicts were perceived to undermine efforts to encourage children's healthy eating habits. Intergenerational feeding conflicts should also be understood within broader cultural and historical contexts. Grandparents' tendency to encourage children to eat more may reflect affection and caregiving responsibility, but may also be shaped by earlier experiences of food scarcity and older understandings of healthy child growth, in which eating more or gaining weight could be interpreted as signs of good care (Jiang et al. 2007; Li et al. 2015). These beliefs may conflict with contemporary understandings of healthy eating in China, which increasingly emphasise dietary diversity, balanced meals, adequate intake of vegetables, fruits, dairy and whole grains, and limiting foods high in salt, sugar and oil (Yang et al. 2018). This shift may partly explain why parents in the present study experienced tension when trying to promote balanced eating while grandparents emphasised fullness, appetite or visible weight gain. However, feeding challenges can also be mitigated by fostering strong support within the family, particularly through supportive co‐parenting (Galvez Espinoza et al. 2022). Some parents acknowledged grandparents helped relieve their feeding burden. These findings suggest that reducing conflict and strengthening collaboration within the family system may be important for creating a supportive feeding environment.

At the external level, consistent with Ecological Systems Theory, parents' support needs reflected the importance of educational, healthcare and information systems in shaping feeding practices. Kindergarten teachers and HCPs also noted that parents' feeding practices could be improved with access to resources, professional guidance, and relevant training opportunities. This suggests a clear need for training programmes, ideally with multiple components (e.g., sessions, personalised feedback, and emotional support), for parents. Consistent with existing research (Frosch et al. 2021), when parents feel supported and informed, they are better equipped to manage feeding challenges. Future interventions to support parental feeding practices should be accessible, evidence‐based, foster collaboration between families, educators, and healthcare providers, and address cultural norms that influence feeding beliefs and behaviours. These support needs also suggest a potential equity dimension, as parents may differ in their ability to access reliable information, professional advice, and training opportunities depending on their work conditions, socioeconomic resources, and connection to health or early education services (Story et al. 2008; Wu et al. 2014). Embedding such support within routine health and early education services may strengthen implementation and better equip parents to foster child healthy eating habits for positive long‐term outcomes.

This study has several limitations. First, as this qualitative study was conducted in China, the findings may not be generalisable to other countries with different cultural contexts. Second, although efforts were made to recruit a diverse sample across socioeconomic and educational backgrounds, the single‐city setting may limit the transferability. Therefore, the findings should be interpreted in light of the specific study setting rather than as representative of all Chinese family feeding practices. Third, although multiple stakeholders' perspectives were included, the study did not involve grandparents, who often serve as secondary caregivers in Chinese families. Therefore, findings related to grandparental involvement reflect the accounts of parents, teachers and HCPs rather than grandparents' own perspectives. Future research should directly explore grandparents' perspectives on feeding practices. Lastly, although purposive sampling was used to include parents with diverse educational and socioeconomic backgrounds, the study was not designed to systematically compare feeding experiences across demographic subgroups or to examine structural and socioeconomic inequalities in depth. Future research could explore how parental education, household income, employment conditions, caregiving arrangements, socioeconomic resources, and service accessibility shape parents' feeding practices and support needs.

## 5 Conclusion

This study highlights the complex and evolving nature of parental feeding practices in early childhood among participating families in China. Drawing on perspectives from parents, teachers, and HCPs, the findings suggest that parents actively seek strategies to manage feeding and attempt to enhance their practices by acquiring knowledge, reflecting on their interactions with their child, and engaging support networks. However, they often struggle due to time pressures, intergenerational conflicts and inconsistent guidance. Future interventions in similar contexts should prioritise accessible, evidence‐based resources, promote collaboration among families, educators, and clinics, and address cultural influences on feeding. Integrating support into routine health and education services may enhance implementation and help parents encourage healthy eating in preschoolers.

## Author Contributions

J.W. led the conceptualisation and project administration, investigated and conducted the research, performed the formal analyses, managed data, resources, software and visualisations, and wrote and refined the original draft. Y.‐S.C. and K.W. contributed to the study design, supervised the research, and reviewed and revised the manuscript. Y.C. supervised the research, reviewed and revised the manuscript. X.W. contributed to investigation, data curation, and formal analysis. All authors reviewed and approved the final manuscript.

## Funding

The authors received no funding for this work.

## Conflicts of Interest

The authors declare no conflicts of interest.

## Supporting information


Supporting File

## Data Availability

The data that support the findings of this study are available on request from the corresponding author. The data are not publicly available due to privacy or ethical restrictions.
