# Supplementary material for: “Taking the Snail for a Walk”: A Multi‐Stakeholder Qualitative Study Exploring Chinese Parents' Feeding Experiences With Preschool Children
Source: Matern Child Nutr. 2026 Jul 28;22(3):e70235. doi: 10.1111/mcn.70235 (PMC13412052; doi:10.1111/mcn.70235)
Supplement: Supplementary file 1 — Supporting File [file MCN-22-e70235-s001.doc]

**Supplementary Materials**

**Table S**1 Summary of the underlying theoretical assumptions in this study

| **Paradigm element** | **Theoretical assumption** | **Definition** | **Application in this study** |
| --- | --- | --- | --- |
| Epistemology | Constructivist | Constructivist epistemology posits that knowledge emerges from the interaction between the participant's experiences and the researcher's interpretations, as well as their engagement with the phenomenon (Madill et al., 2000). | The researchers prioritise meaningfulness over frequency in the codes, elucidating the meaning behind the codes. |
| Orientation | Experiential | Experiential orientation prioritises the examination of how a given phenomenon may be experienced by the participant (Braun & Clarke, 2013). | The researchers recognise that participants' verbal reports are interpretations of their internal personal feelings. |
| Analysis | Inductive and deductive | Inductive coding generates codes directly from the data, allowing themes to emerge organically during analysis; deductive coding applies codes derived from prior knowledge, personal experiences, and pre-existing theoretical frameworks to the data. | The researchers use inductive to generate codes directly from the data and employ deductive coding to guide data coding and explore and determine the final themes with prior theories, knowledge, and experiences. |
| Themes | Latent | Latent themes interpret what is hidden within the text to understand the implied meaning (Kleinheksel et al., 2020). | The researchers analyse beyond the transcripts, using themes to connect meanings relevant to the research question. |

**Table S2** Consolidated criteria for reporting qualitative studies (COREQ): 32-item checklist

| **Topic** | **Item No.** | **Guide questions/description** | **Reported in**  **Section** |
| --- | --- | --- | --- |
| **Domain 1: Research team and reﬂexivity** | | | |
| *Personal Characteristics* | | | |
| Interviewer/facilitator | 1 | Which author/s conducted the interview or focus group? | 2.3 Data collection |
| Credentials | 2 | What were the researcher’s credentials? E.g. PhD, MD | 2.6 Reflexivity |
| Occupation | 3 | What was their occupation at the time of the study? | 2.3 Data collection |
| Gender | 4 | Was the researcher male or female? | 2.6 Reflexivity |
| Experience and training | 5 | What experience or training did the researcher have? | 2.6 Reflexivity |
| *Relationship with participants* | | | |
| Relationship established | 6 | Was a relationship established prior to study commencement? | 2.6 Reflexivity |
| Participant knowledge of the interviewer | 7 | What did the participants know about the researcher? e.g. personal goals, reasons for doing the research | 2.6 Reflexivity |
| Interviewer characteristics | 8 | What characteristics were reported about the interviewer/facilitator? e.g. Bias, assumptions, reasons and interests in the research topic | 2.6 Reflexivity |
| **Domain 2: study design** | | | |
| *Theoretical framework* | | | |
| Methodological orientation and Theory | 9 | What methodological orientation was stated to underpin the study? e.g. grounded theory, discourse analysis, ethnography, phenomenology, content analysis | 2.1 Study design |
| *Participant selection* | | | |
| Sampling | 10 | How were participants selected? e.g. purposive, convenience, consecutive, snowball | 2.3 Data collection |
| Method of approach | 11 | How were participants approached? e.g. face-to-face, telephone, mail, email | 2.3 Data collection |
| Sample size | 12 | How many participants were in the study? | 3.1 Sample characteristics |
| Non-participation | 13 | How many people refused to participate or dropped out? Reasons? | 3.1 Sample characteristics |
| *Setting* | | | |
| Setting of data collection | 14 | Where was the data collected? e.g. home, clinic, workplace | 2.3 Data collection |
| Presence of non-participants | 15 | Was anyone else present besides the participants and researchers? | N/A |
| Description of sample | 16 | What are the important characteristics of the sample? e.g. demographic data, date | 3.1 Sample characteristics |
| *Data collection* | | | |
| Interview guide | 17 | Were questions, prompts, guides provided by the authors? Was it pilot tested? | Table S3 |
| Repeat interviews | 18 | Were repeat interviews carried out? If yes, how many? | No |
| Audio/visual recording | 19 | Did the research use audio or visual recording to collect the data? | 2.3 Data collection |
| Field notes | 20 | Were ﬁeld notes made during and/or after the interview or focus group? | 2.3 Data collection |
| Duration | 21 | What was the duration of the interviews or focus group? | 3.1 Sample characteristics |
| Data saturation | 22 | Was data saturation discussed? | 2.2 Settings, recruitment and participants |
| Transcripts returned | 23 | Were transcripts returned to participants for comment and/or correction? | No |
| **Domain 3: analysis and findings** | | | |
| *Data analysis* | | | |
| Number of data coders | 24 | How many data coders coded the data? | 2.4 Data analysis |
| Description of the coding tree | 25 | Did authors provide a description of the coding tree? | NA |
| Derivation of themes | 26 | Were themes identiﬁed in advance or derived from the data? | 2.4 Data analysis |
| Software | 27 | What software, if applicable, was used to manage the data? | 2.4 Data analysis |
| Participant checking | 28 | Did participants provide feedback on the ﬁndings? | No |
| *Reporting* | | | |
| Quotations presented | 29 | Were participant quotations presented to illustrate the themes/ﬁndings? Was each quotation identiﬁed? e.g. participant number | Table 2 |
| Data and ﬁndings consistent | 30 | Was there consistency between the data presented and the ﬁndings? | 3.2 Thematic findings |
| Clarity of major themes | 31 | Were major themes clearly presented in the ﬁndings? | 3.2 Thematic findings |
| Clarity of minor themes | 32 | Is there a description of diverse cases or discussion of minor themes? | Table 2 |

Developed from: Tong A, Sainsbury P, Craig J. Consolidated criteria for reporting qualitative research (COREQ): a 32-item checklist for interviews and focus groups. *International Journal for Quality in Health Care*. 2007. Volume 19, Number 6: pp. 349 – 35

**Table S3 Interview guide**

| ***Instructions*** |
| --- |
| - Explain the objectives of this study to the interviewee. |
| - Be sure that informed consent has been obtained for the interview and audio recording. |
| - The interview guide is divided into three sections; A, B and C. Section A is for parents of preschool children, section B is for kindergarten teachers and section C is for HCPs. Use the appropriate section for each participant. |
| ***Section 1: For parents of preschool children (n = 35, semi-structured Interviews)*** |
| **Feeding Practices** |
| - Please tell me about your views on feeding practices (If you have two or more preschool children, please choose one child to focus on during the interviews based on what you feel is most relevant to your experiences).   Prompts: feeding practices (factors, needs, difficulties, specific feeding, Chinese culture, family environment) |
| - How do you think your current feeding practices are? |
| - Which aspects of your feeding practices do you find challenging or think need improvement? Why? How did you handle these feeding issues? |
| - Which aspects of your feeding practices are you most satisfied with? Could you share how you achieved these outcomes? |
| - What are your needs or expectations for knowledge, skills or other resources related to adopting appropriate feeding practices or optimising your feeding practices? |
| - Which potential barriers and enablers for the implementation of this intervention? |
| ***Section 2: For kindergarten teachers (n = 16, three focus groups)*** |
| **Feeding practices** |
| - Please tell me about your views on parental feeding practices.   Prompts: specific feeding practices, child eating, growth.   - Which problems do you think of in parental feeding? What do you think are the reasons behind this? |
| - Which aspects of feeding do you believe parents are doing well? Why? |
| - Do you provide any assistance to parents regarding child feeding practices? If so, in which areas? |
| - From your perspective, what can be done to optimise parental feeding practices or provide support for parents dealing with feeding issues? |
| ***Section 3: For HCPs (n = 11, semi-structured interviews)*** |
| **Feeding practices** |
| - Please tell me about your views on parental feeding practices.   Prompts: specific feeding practices, child eating, growth. |
| - Which problems do you think of in parental feeding? What do you think are the reasons behind this? |
| - Which aspects of feeding do you believe parents are doing well? Why? |
| - Do you provide any assistance to parents regarding child feeding practices? If so, in which areas? |
| - From your perspective, what can be done to optimise parental feeding practices or provide support for parents dealing with feeding issues? |

**References**

Braun, V., and V. Clarke. 2013. Successful Qualitative Research: A Practical Guide for Beginners. London: SAGE Publications Ltd.

Kleinheksel, A. J., N. Rockich-Winston, H. Tawfik, and T. R. Wyatt. 2020. “Demystifying Content Analysis.” American Journal of Pharmaceutical Education 84, no. 1: 7113. <https://doi.org/10.5688/ajpe7113>

Madill, A., A. Jordan, and C. Shirley. 2000. “Objectivity and Reliability in Qualitative Analysis: Realist, Contextualist and Radical Constructionist Epistemologies.” British Journal of Psychology 91, no.  1: 1–20. https://doi.org/10.1348/000712600161646
